# Diffuse large B-cell lymphoma with combined *TP53* mutation and *MIR34A* methylation: Another “double hit” lymphoma with very poor outcome?

**DOI:** 10.18632/oncotarget.1877

**Published:** 2014-03-31

**Authors:** Fazila Asmar, Christoffer Hother, Gorjan Kulosman, Marianne Bach Treppendahl, Helene Myrtue Nielsen, Ulrik Ralfkiaer, Anja Pedersen, Michael Boe Møller, Elisabeth Ralfkiaer, Peter de Nully Brown, Kirsten Grønbæk

**Affiliations:** ^1^ Department of Hematology, Rigshospitalet, Copenhagen, Denmark,; ^2^ Department of Pathology, Rigshospitalet, Copenhagen, Denmark,; ^3^ Department of Pathology, Odense University Hospital, Odense, Denmark.

**Keywords:** Epigenetic changes, DNA methylation, microRNA, Tumor suppressors, non-Hodgkin lymphoma

## Abstract

MiR34A, B and C have been implicated in lymphomagenesis, but information on their role in normal CD19+ B-cells (PBL-B) and *de novo* diffuse large B-cell lymphoma (DLBCL) is limited.

We show that in normal and activated B-cells miR34A-5p plays a dominant role compared to other miR34 family members. Only miR34A-5p is expressed in PBL-B, and significantly induced in activated B-cells and reactive lymph nodes. In PBL-B, the *MIR34A* and *MIR34B/C* promoters are unmethylated, but the latter shows enrichment for the H3K4me3/H3K27me3 silencing mark.

Nine de novo DLBCL cases (n=150) carry both *TP53* mutation and *MIR34A* methylation (“double hit”) and these patients have an exceedingly poor prognosis with a median survival of 9.4 months (*P*<0.0001), while neither *TP53* mutation, *MIR34A* or *MIR34B/C* promoter methylation alone (“single hit”) influence on survival. The *TP53/MIR34A* “double-hit” is an independent negative prognostic factor for survival (*P*=0.0002). In 2 DLBCL-cell lines with both *TP53* mutation and promoter methylation of *MIR34A*, miR34A-5p is upregulated by 5-aza-2'deoxycytidine. Thus, the *TP53/MIR34A* “double hit” characterizes a very aggressive subgroup of DLBCL, which may be treatable with epigenetic therapy prior to or in combination with conventional immunochemotherapy.

## INTRODUCTION

Diffuse large B-cell lymphoma (DLBCL) comprise a variety of morphological and clinical phenotypes, resulting from miscellaneous, underlying molecular defects. Gene expression profiles and their immunohistochemical surrogate markers have been used to classify DLBCL into prognostic subgroups [1, 2], still, diversity in biology and clinical outcome exists within the individual categories. Only few genetic or epigenetic defects have been identified as prognostic markers. The data on *TP53* mutation alone as a prognostic factor for survival in DLBCL have been inconsistent, however, a number of studies have shown that dual disruption of the p16/Rb and the ARF/p53 pathway, e.g. deletion of the *INK4A/ARF* locus, are strong negative prognostic factors for survival [3–6]. Likewise, the combined translocations of oncogenic *MYC* and anti-apoptotic *BCL-2* or *BCL-6* (so-called “double hit” lymphoma) are associated with exceedingly poor prognosis [7, 8].

Most previous studies of tumor suppressor pathways in DLBCL have focused on the disruption of the coding sequences of genes. However, epigenetic alterations and aberrant expression of microRNAs (miRs) may be equally important for growth control. As for protein encoding genes, the transcription of miR genes may be inactivated by promoter hypermethylation [9, 10].

The members of the miR34 family (miR34A, miR34B, and miR34C) have been recognized as tumor suppressors, and implicated in a variety of cellular processes that control carcinogenesis including cell cycling, apoptosis, somatic cell reprogramming and metastasis [11–14]. It has been shown that the p53-miR34 axis may be another link between the ARF/p53 pathway, the p16/Rb pathway and MYC regulated pathways. In a complex circuit, p53 promotes transcription of *MIR34A* and *MIR34B/C* , and the miR34s in turn act as mediators of p53 signaling [15, 16]. In addition, miR34s inhibit MYC and several proto-oncogens that counteract the p16/Rb tumor suppressor pathway [17–19]. These observations place the miR34s at the center of cell cycle and apoptosis regulation, and loss of miR34 expression has been associated with poor response to therapy.

Several studies have implicated both miR34A [20–22] and miR34B/C [15, 23, 24] in lymphoproliferative malignancies. In CLL, the p53-miR34A and p53-miR34B/C axes have been investigated in detail. Low expression levels of miR34A correlate to *TP53* mutation or 17p deletion, and has negative prognostic impact on both treatment free survival [20] and survival of previously treated patients [22]. MiR34B and miR34C have been shown to act with miR15, miR16, p53 and ZAP70 [15]. In multiple myeloma the *MIR34B/C* cluster is downregulated by promoter methylation in a large proportion of cases at relapse [24]. In low-grade gastric MALT type lymphoma downregulation of miR34A is involved in the transformation to DLBCL, by deregulation of the oncogene FOXP1 [21]. However, in *de novo* DLBCLs, the miR34s have not previously been investigated, and in spite of their implication in several other lymphoproliferative malignancies, little is known about the role of the miR34s in normal B-cells.

Here, we investigated the expression of miR34A, miR34B and miR34C in normal and reactive B-cells. Given that *MIR34A* and *MIR34B/C* locate to regions of allelic loss in DLBCL (1p36.23 and 11q23.1, respectively) [4, 25, 26], and the importance of the miR34 targets in DLBCL pathogenesis, we also investigated a large panel of newly diagnosed cases of DLBCLs for *MIR34A* and *MIR34B/C* promoter methylation, *TP53* mutational status, clinical presentation patterns, and outcome.

## RESULTS

Several studies have shown the implication of the miR34s in CLL and multiple myeloma, and a single study implicated miR34A in the transformation of gastric MALT-type lymphomas to DLBCL [20–24]. However, the role of the miR34s in *de novo* DLBCLs has not been analysed in detail, and little is known about the role of the miR34s in normal B-cells.

p53 has been shown to directly bind to and regulate the transcription of both miR34A and miR34B/C [16], and we speculated whether these molecules were involved in DLBCL lymphomagenesis in a mutually exclusive manner, as it was previously suggested in CLL [20]. DLBCL consists of mixtures of malignant B-cells and reactive cells, which complicates the measurement of miR34 expression. Therefore, we initially determined the expression of the individual miR34 family members in PBL-B and in reactive lymph nodes. Since these were differentially expressed, we next investigated miR34 regulation at the DNA level in DLBCL to avoid mis-interpretation of miR expression signals from infiltrating cells. Thus, we examined the components of the p53-miR34 axis including *TP53* mutational status and hypermethylation of the *MIR34A* and *MIR34B/C* promoters in a panel of 150 newly diagnosed DLBCL and in 6 DLBCL cell lines, and compared these data to those obtained from PBL-B and reactive lymph nodes.

### MiR34A, miR34B, and miR34C expression and regulation in non-cancerous B-cells

**Figure 1:**
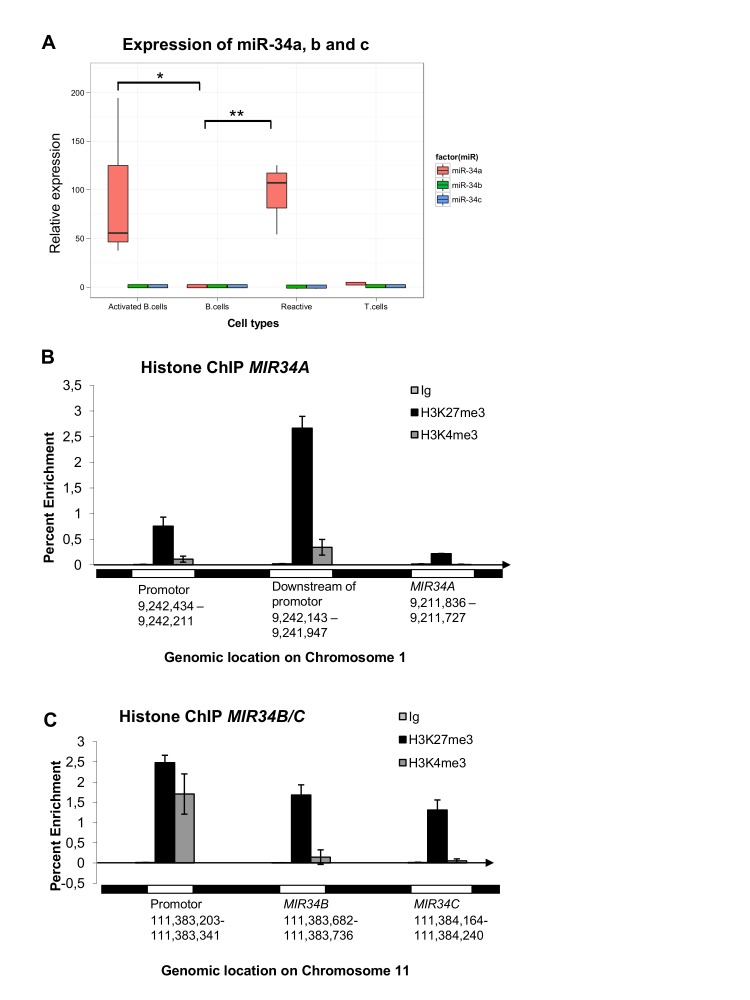
MiR34A, miR34B, and miR34C expression and regulation in normal B-cells, T-cells and reactive lymph nodes (A) Normal peripheral blood CD19+ B-lymphocytes (PBL-B) have an unmethylated *MIR34A* promoter and show low expression of miR34A-5p, which, however, is significantly upregulated in reactive lymph nodes (*P*<0.001) and *in vitro* activated B-cells (*P*<0.017). MiR34A-5p is not expressed in normal CD3+ T-cells. The *MIR34B/C* promoter is unmethylated and miR34B and miR34C is not expressed in normal or *in vitro* activated B-cells, T-cells or reactive lymphnodes. (B) No enrichment of the H3K27me3 or H3K4me3 marks was observed at the TSS of *MIR34A*. (C) The downregulation of miR34B/C in PBL-B may be due to enrichment for the bivalent H3K27me3/H3K4me3 silencing mark at the *MIR34B/C* promoter.

We began our investigations by examining the methylation and expression status of miR34A-5p, miR34A-3p, miR34B-5p, miR34B-3p, miR34C-5p and miR34C-3p in PBL-B from random donors and in reactive lymph nodes. DNA from normal PBL-B was unmethylated at both the *MIR34A* and *MIR34B/C* promoters. While miR34A-5p was expressed at low levels in normal PBL-B, no expression of miR34A-3p, miR34B-5p, miR34B-3p, miR34C-5p, and miR34C-3p could be detected in PBL-B (Ct values>35 cycles)). In reactive lymph nodes the expression of miR34A-5p was induced in average 70 fold (*P*<0.001), while no significant induction was observed for the rest of the investigated miRs, including the homologous miR34B-5p and miR34C-5p, indicating that the assay is highly specific to miR34A-5p (Figure 1A).

Since the reactive lymph nodes consist of a mixture of cells, we next stimulated PBL-B *in vitro* by LPS and IL-1α activation and observed significant induction of miR34A-5p (*P*= 0.017, t-test), but not of miR34B/C. In addition, normal CD3+ T cells showed no expression of miR34A, indicating that the response we observe in reactive lymph nodes most likely originate from activated B-cells (Figure 1A).

Given the low expression of miR34s in normal PBL-B, we investigated if miR34A, B and -C were downregulated by other epigenetic mechanisms. Chromatin immunoprecipitation (ChIP) at the *MIR34A*- and the *MIR34B/C* promoters showed a relative enrichment (>1,0 of input DNA) for the bivalent H3K27me3/H3K4me3 silencing mark at the *MIR34B/C* promoter (Figure 1B).

Taken together, these results indicate that in PBL-B both miR34A and miR34B/C are expressed at low levels. The miR34B/C cluster is downregulated by polycomb, and in contrast to miR34A, does not seem to be reactivated upon stimulation.

### MiR34A, -B, and -C expression is associated with promoter methylation in DLBCL cell lines and can occur in combination with TP53 mutations

**Figure 2:**
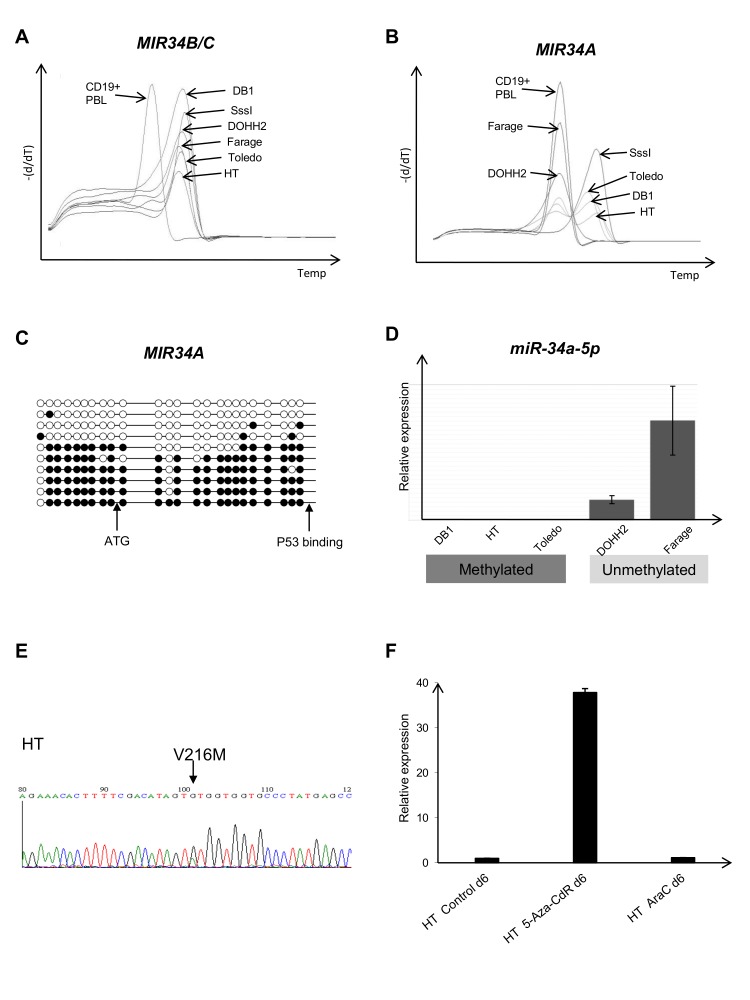
MIR34A, MIR34B/C and TP53 regulation in lymphoma cell lines (A) MS-MCA showing that all cell lines are methylated at the *MIR34B/C* promoter in the DLBCL cell lines. (B) In the DLBCL cell lines Toledo, DB1 and HT, the *MIR34A* promoter shows a biphasic methylation pattern (methylated and unmethylated), while Farage and DOHH2 are completely unmethylated. (C) Bisulfite sequencing of the *MIR34A* promotor region showing methylation of ~50% of the *MIR34A* alleleles, methylated cytosines, unmethylated cytosines. (D) *MIR34A* promoter methylation correlates with expression. (E) Direct sequencing showing the concomitant *TP53* mutation (V216M) in the *MIR34A/B/C* methylated cell line HT (F) Upregulation of miR34A in the *MIR34A* methylated and *TP53* mutant cell line HT after treatment with the hypomethylating agent 5-aza-2'deoxycytidine (5-aza-CdR 0.5uM). No induction of miR34A was observed after treatment with cytosine arabinoside (araC 20nM).

We next investigated the methylation status of the *MIR34A* and *MIR34B/C* promoters in DLBCL-cell lines, and compared these data with *TP53* mutational status. In all DLBCL cell lines the *MIR34B/C* promoter was methylated (Figure 2A) and miR34B/C not expressed (data not shown). By contrast, only the Toledo, DB1, HT, and U2932 cell lines showed a biphasic melting curve at the *MIR34A* promoter indicating that both methylated and unmethylated alleles were present, while the Farage and DOHH2 cell lines had a completely unmethylated *MIR34A* promoter (Figure 2B and Supplemental Figure 1). Bisulfite genomic sequencing of the HT-cell line showed that ~50 % of the *MIR34A* alleles were fully methylated and, ~50% were fully unmethylated , suggesting this could be caused by monoallelic methylation, or alternatively, be indicative of an unmethylated subclone (Figure 2C). While the completely unmethylated Farage and DOHH2 cells showed an expression level of miR34A-5p that was comparable to that of reactive lymph nodes, no expression of miR34A-5p was detected in the cell lines with both methylated and unmethylated alleles (~total 2 copies pr. cell as measured by quantification of a miR34A-5p mimic) (Figure 2D). These data indicate that the observed level of methylation at the *MIR34A* promoter is associated with blocked transcription of *MIR34A*.

Mutation analysis of exons 5-9 of the *TP53* gene in the six DLBCL cell lines revealed a G to A transition causing the V216M hotspot mutation in the HT cell line (Figure 2E) and the C176Y mutation in U2932 was confirmed [27]. Surprisingly, both cell lines also showed both *MIR34A* and *MIR34B/C* promoter hypermethylation. Treatment of the *TP53* mutant/*MIR34A/B/C* promoter methylated cell lines HT and U2932 with 5-aza-CdR showed that miR34A-5p (but none of the other miRs) are upregulated irrespective of the *TP53* mutation (Figure 2F and Supplemental Figure 1 for U2932). Treatment with cytosine arabinoside did not induce miR34A-5p.

### Coordinated promoter methylation of MIR34A and MIR34B/C in primary DLBCL

**Figure 3:**
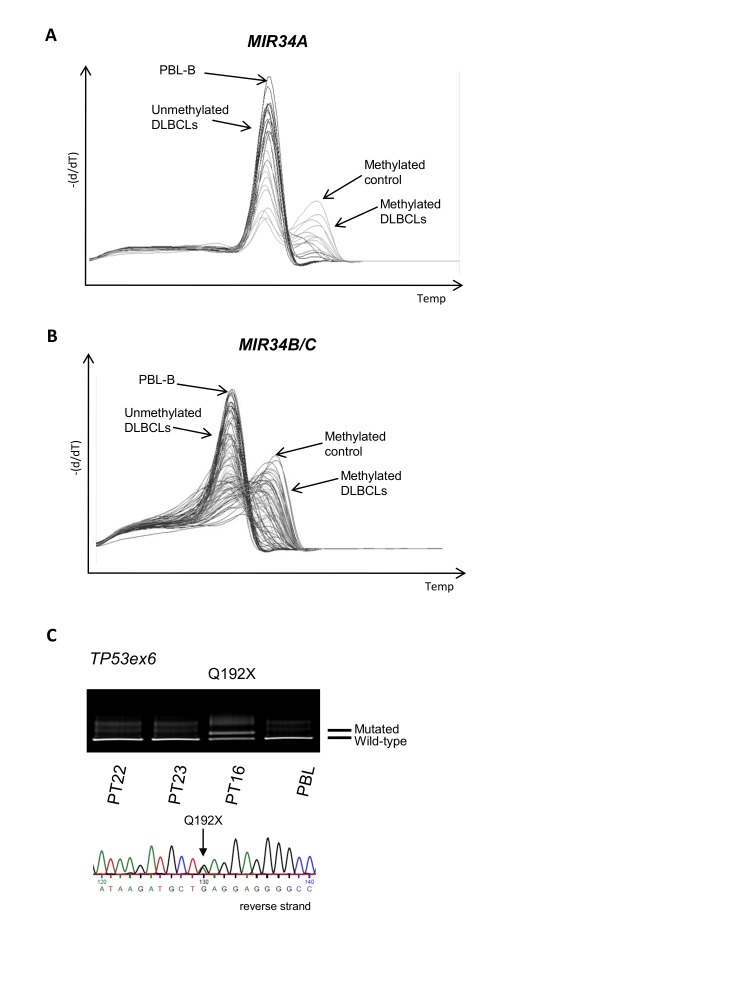
MIR34A, MIR34B/C and TP53 deregulation in primary DLBCLs (A) *MIR34A* methylation in primary DLBCL by MS-MCA. (B) *MIR34B/C* methylation in primary DLBCL (MS-MCA). (C) Representative image of DGGE gel and Sanger sequence of *TP*53 mutation in primary DLBCL.

To investigate whether these combined molecular alterations also occur *in vivo*, we next examined a panel of 150 primary DLBCL samples for promoter methylation of *MIR34A* and *MIR34B/C* by MS-MCA. *MIR34A* was methylated in 42 cases (28%) (Figure 3A), and *MIR34B/C* was methylated in 116 (78%) cases (Figure 3B). All except two of the cases with methylation of *MIR34A* also carried methylation of the *MIR34B/C* promoter (*P*=0.001, Fisher's exact test), suggesting that the methylation of these miRs may be interdependent and occur in coordinated manner. The *MIR34A* methylated cases did not differ significantly from the *MIR34A* unmethylated cases with respect to age, sex, clinical stage, LDH, performance score or IPI (Table 1), which was also the case for *MIR34B/C* methylated vs. unmethylated cases (data not shown).

### TP53 and MIR34A mutations, and the MDM2 SNP309 GG genotype in DLBCL

DGGE-based screening of exons 5-9 of the *TP53* gene revealed mutations in 24 out of 150 (16%) of the primary DLBCL cases (Figure 3C). These mutations comprised both missense and non-sense mutations (Supplemental Table 2), and were distributed throughout the investigated exons. One case carried two mutations suggesting they may be biallelic. No mutations or SNPs were detected in the *MIR34A* expressed sequence, although 2 SNPs have previously been reported in the region (*www.genome.ucsc.edu*). Since studies in CLL suggest that the *MDM2* SNP309 GG genotype associate with low miR34A expression and poor prognosis, we investigated the role of this SNP in a subset (120) of these primary DLBCLs, but observed no prognostic impact (see Supplemental Material).

### A subset of de novo DLBCL carries concomitant methylation of MIR34A and TP53 mutation

By combining the above data we identified a subset of nine primary DLBCL cases that carried combined *TP53* mutation and *MIR34A* promoter methylation at diagnosis. The *TP53* mutations were both loss of function mutations (4) and missense mutations in the DNA binding domain (5) (Supplemental Table 2). A total of 9 of the 24 (38%) *TP53* mutant cases also had *MIR34A* methylation and 9 of the 42 (21%) *MIR34A* methylated cases also had *TP53* mutation (*P*=0.19), indicating that these alterations do occur independently of each other. These data support our findings from the cell lines, that a subset of DLBCL has combined loss of p53 and miR34A function. We observed that the *MIR34A* methylated and *TP53* mutated patients were associated with aggressive disease with Ann Arbor stage III-IV (*P*=0.030), elevated LDH level (*P*=0.015), and IPI group III-IV (*P*=0.023). In the cohorte of patients treated with Rituximab the *MIR34A* methylated and *TP53* mutated cases were also significantly associated with Ann Arbor stage III-IV (*P*=0.025), elevated LDH level (*P*=0.002), and IPI group III-IV (*P*=0.027) (Table 1).

**Table 1a:** Clinical characteristics of all DLBCL patients at time of diagnosis (n=150, 62 Rituximab and 88 non-Rituximab treated DLBCL patients)

	n	*TP53*wt and *MIR34A* unmeth	*TP53*mut or *MIR34A* meth	*TP53*mut and *MIR34A* meth	*P*-value
*Mean Age (yrs)*	150	59	63	65	0.270
*Sex*	147				0.495
F		39	23	4	
M		55	21	5	
*Stage*	145				0.030
I+II		46	24	1	
III+IV		48	18	8	
*Performance score*	144				0.129
0+1		79	35	5	
2+3		14	6	4	
*S-LDH*	139				0.015
Normal		46	26	1	
Elevated		45	14	7	
*IPI*	144				0.023
L+LI		65	30	2	
H+HI		25	10	6	

**Table 1b:** Clinical characteristics of Rituximab treated DLBCL patients at time of diagnosis (n=62)

	n	*TP53*wt and *MIR34A* unmeth	*TP53*mut or *MIR34A* meth	*TP53*mut and *MIR34A* meth	*P*-value
*Mean Age (yrs)*	62	62	63	60	0.850
*Sex*	62				0.208
F		18	6	3	
M		23	9	3	
*Stage*	62				0.025
I+II		18	8	0	
III+IV		23	7	6	
*Performance score*	62				0.226
0+1		38	14	4	
2+3		3	1	2	
*S-LDH*	62				0.002
Normal		26	11	0	
Elevated		15	4	6	
*IPI*	62				0.027
L+LI		30	10	1	
H+HI		11	5	5	

### Overall Survival

**Figure 4:**
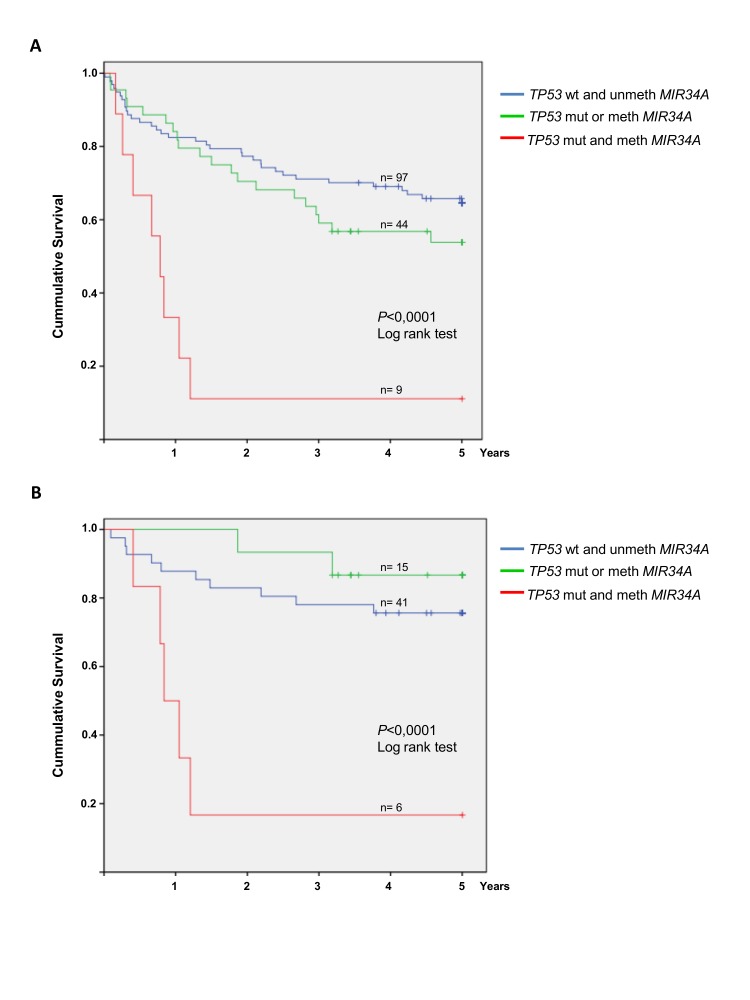
Overall survival of DLBCL patients with and without MIR34A methylation and TP53 mutation Patients are divided into 3 groups: Patients with no alterations of *TP53* or *MIR34A*, patients with *either MIR34A* methylation *or TP53* mutation only, and patients with concomitant *MIR34A* methylation and *TP53* mutation. (A) 5 year overall survival for the three groups in the entire cohorte (n=150). The group with concomitant *MIR34A* methylation and *TP53* mutation (n=9) shows significantly poor overall survival (*P*<0.0001). (B) Among the Rituximab treated patients (n=62) concomitant *MIR34A* methylation and *TP53* mutation (n=6) leads to significantly inferior survival as well (*P*<0.0001).

We initially analyzed the prognostic impact on overall survival of each individual molecular alteration. The Kaplan-Meier estimate of overall survival for cases with methylation of *MIR34B/C* did not differ from cases with unmethylated *MIR34B/C* (*P*=0.803). Cases with *TP53* mutations had significantly poorer survival (*P*=0.004), whereas cases with *MIR34A* methylation showed a tendency towards poorer survival (*P*=0.077). However, the 9 cases with disruption of both *TP53* and *MIR34A* showed an exceedingly poor survival of only median 9.4 months (*P*<0.0001) (Figure 4A). Interestingly, 6 of the 9 “double-hit” cases were treated with Rituximab in addition to combination chemotherapy, which did not seem to improve survival (Figure 4B).

To compare the relative impact on survival of either *TP53* mutation or *MIR34A* methylation alone to that of concomitant *TP53* mutation and *MIR34A* methylation, we performed a multivariate Cox regression analyses. Baseline risk factors included in the model were: age, LDH, Ann Arbor stage, performance status and IPI. The model revealed that *TP53* mutation or *MIR34A* methylation alone are not independent factors for survival. However, combined *MIR34A* methylation and *TP53* mutation is an independent factor for survival both in the entire cohorte (*P* = 0.0002) and in the Rituximab treated patients (*P*=0.021). Furthermore, elevated LDH, age, performance status and lack of Rituximab are independent prognostic factors in the entire cohorte, while only LDH and age retain negative prognostic impact in Rituximab treated patients (Table 2).

**Table 2:** 

Multivariate analysis	Hazard ratio	95% Hazard Ratio Lower	Confidence Limits Upper	*P*
Rituximab treated patients (n=62)				
*MIR34A* unmeth and *TP53*wt	1.000			
*MIR34A* meth or *TP53*mut	0.550	0.120	2.530	0.4433
*MIR34A* meth and *TP53*mut	4.364	1.245	15.302	0.0213
LDH (normal/elevated)	3.699	1.081	12.663	0.0372
Age	1.044	1.008	1.081	0.0156
All patients (n=150)				
*MIR34A* unmeth and *TP53wt*	1.000			
*MIR34A* meth or *TP53*mut	1.449	0.800	2.800	0.2035
*MIR34A* meth and *TP53*mut	6.518	2.461	17.264	0.0002
LDH (normal/elevated)	3.094	1.621	5.903	0.0006
Performance status (0-1/2-4)	2.135	1.151	3.963	0.0162
Age	1.044	1.023	1.064	<.0001
Immunotherapy (No/yes)	2.449	1.300	4.611	0.0055

**Figure 5:**
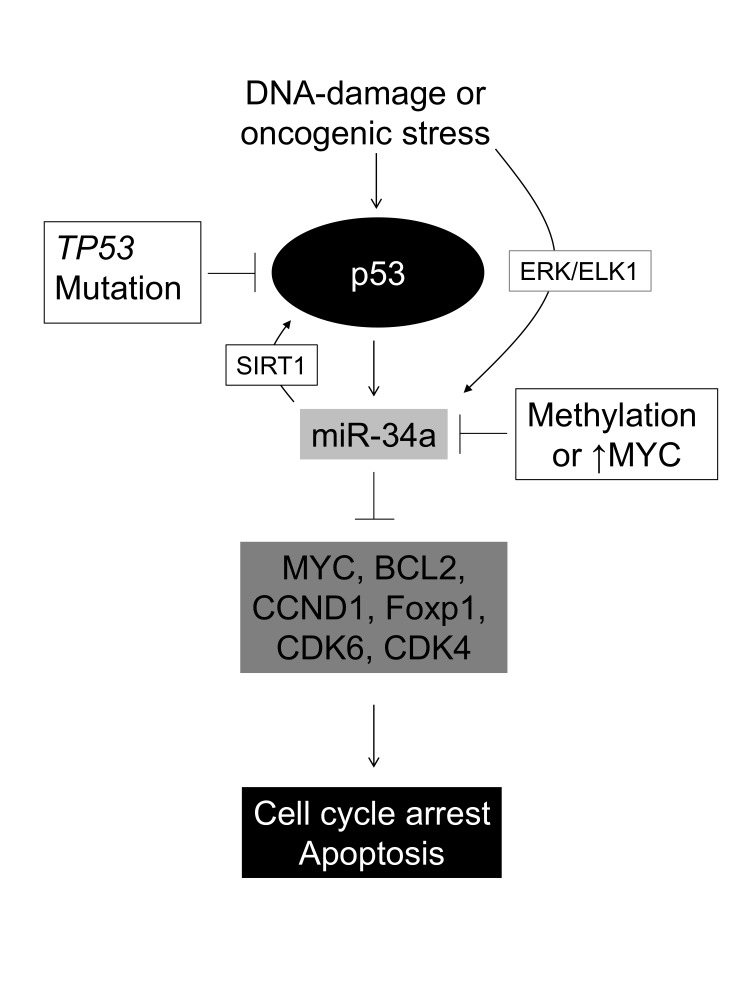
A schematic model of the p53-miR34 axis Oncogenic stress or DNA damage may activate p53 leading to cell cycle arrest, senescense and/or apoptosis. This is mediated via its downstream effectors miR34A -B and -C, which in turn downregulate known DLBCL proto-oncogenes. Our data indicate that DLBCL cells with concomitant *TP53* mutation and *MIR34A* methylation have a growth advantage compared to “single-hit” tumors, suggesting that in DLBCL miR-34A may also be upregulated independently of p53 by oncogenic stress, probably via the ERK/ELK pathway as suggested by Christoffersen et al [17].

## DISCUSSION

Within the last decade, miRs have been recognized as important players in tumorigenesis and many miRs may serve as markers of disease. In DLBCL, miRs may be used in disease classification and as predictors of outcome [28–30], and some miRs may be directly involved in DLBCL pathogenesis [31, 32].

Disruption of the individual miRs in the miR34 family has been demonstrated in a variety of cancers, and has got attention due to the direct involvement in the p53 and MYC pathways. Overexpression of miR34 in p53 deficient cells can reinstate p53 functions such as senescence, apoptosis and cell cycle arrest [16, 33]. The miR34 family inhibits cell cycle progression at the G1/S transition checkpoint by targeting the mRNAs of several proto-oncogenes that act in the p16 and ARF/p53 pathways including N- and C-MYC, CDK6, CDK4, and CCND1 [16–19, 34]. Furthermore, miR34A may create a positive feedback loop on p53 by downregulating the deacetylase SIRT1, leading to p53 acetylation and activation [35], and miR34A has also been shown to target pro-apoptotic BCL-2 for downregulation. The miRs 34B/C have been shown to repress MYC in response to DNA damage [34, 36], and in Burkitts' lymphoma miR34B/C downregulation was suggested to be a more common mechanism for MYC overexpression than *IGH/MYC* translocation [34]. In addition, overexpression of miR34A was shown to disrupt normal B-lymphocyte development by targeting FOXP1 [37], which acts as an oncogene in DLBCL when miR34A is downregulated [21]. Importantly, miR34 mimics have been shown to abrogate growth and induce apoptosis in DLBCL *in vivo* [38].

In the present study we initially analyzed the role of the individual members of the miR34 family in normal PBL-B and reactive lymph nodes. Interestingly, miR34A-5p seems to play by far the most dominant role in normal reactive lymph nodes *in vivo*. In line with other tumor suppressors it is expressed at low levels in normal PBL-B, but is upregulated significantly in activated B-cells and reactive lymph nodes. The high expression of miR34A-5p in reactive cells may be an important limitation to measuring expression of this miR in primary DLBCLs, which are typically infiltrated by reactive B-cells. In normal PBL-B the *MIR34B/C* promoter is enriched for the bivalent silencing mark H3K27me3/H3K4me3, and miR34B and miR34C are not expressed. No significant induction of¨miR34A-3p, miR34B or miR34C was observed during B-cell activation or in reactive lymph nodes. Taken together, these results indicate that among the miR34s, miR34A-5p plays a dominant role in regulating normal B-cell function.

Several *in vitro* studies show that deregulation of miR34s is important in lymphomagenesis [15, 34]. In line with what has been shown for other tumor suppressors, we observe that *MIR34B/C* undergo an epigenetic switch from polycomb mediated gene silencing to permanent gene silencing by DNA methylation during lymphomagenesis [39–41]. In addition, concurrent DNA methylation of *MIR34B/C* is present in all but two of the *MIR34A* methylated DLBCLs. This suggests that under certain circumstances e. g. oncogenic stress, these molecules may potentially compensate for each other, and that a cellular growth advantage is only acquired when all three molecules are efficiently downregulated.

Surprisingly, our DLBCL cell line data shows that methylation of approximately half of the investigated alleles at the reported *MIR34A* TSS [33], is associated with miR34A-5p downregulation. By contrast, cell lines with a completely unmethylated *MIR34A* promoter show a high miR34A-5p expression. A previous study showed that many cancer cell lines carry both methylated and unmethylated *MIR34A* alleles, and that the expression is significantly reduced in cells with both methylated and unmethylated alleles, as compared to cells with completely unmethylated alleles [42]. As opposed to the previous study, we analyzed the miR34A-5p expression in the DLBCL cell lines by real time quantitative PCR, and could show that cells with both methylated and unmethylated *MIR34A* alleles have extremely low miR34A-5p expression (corresponding to ~2 copies of miR34A-5p per cell). Thus, our data suggest that mechanisms different than promoter methylation may keep the unmethylated alleles silenced , which is a subject for further investigation.

Two previous studies focus on the role of *MIR34A* methylation in lymphomagenesis. Analyses of a small sample (32 miscellaneous types of NHL, no DLBCLs analyzed) showed promoter hypermethylation primarily in NK/T-cell NHL [43], and a second study showed that miR34A is downregulated by promoter hypermethylation (4 of 7 analyzed DLBCLs) and/or MYC overexpression during the transformation of gastric MALT type lymphoma to aggressive gastric DLBCL [21]. However, in both studies only a limited amount of cases are investigated and none of them address the prognostic impact of *MIR34A* methylation. For the *MDM2* SNP309 our observations are in line with previous studies [44, 45], which also show that the outcome in DLBCL is independent of the GG-phenotype.

The prognostic role of *TP53* mutations in DLBCL is still debated [3, 44, 46–48] however a recent, large study shows that *TP53* disruption is still a negative prognostic factor for survival after the implementation of Rituximab[48]. In the present, relatively small study (n=62), none of the Rituximab treated patients with *TP53* mutation only had died (Supplemental Figure 2), while all but one of those with concommitant disruption of *TP53* and *MIR34A* died within the first 13 months from diagnosis. The only patient with “double hit” lymphoma that is alive after more than 5 years, is a 38 years old woman, which on revision had a primary mediastinal large B-cell lymphoma, (PMBL). This was the only patient in the “double-hit” group to achieve an etoposide containing regimen (R-CHOEP). Interestingly, PMBL was recently shown to have particularly good outcome when treated with the same drugs (dose-adjusted EPOCH-R) [49].

These data are supported by our multivariate analysis which show that neither methylation of *MIR34A* nor *TP53* mutation alone influence survival , while the *MIR34A/TP53* “doublehit” is an independent factor for survival. This is quite surprising given that studies in CLL have indicated that miR34A acts in concert with p53 and MDM2, and may serve as a surrogate marker for disruption of these molecules. Furthermore, it has been suggested that the apoptosis inducing function of miR34A is p53 dependent [50]. However, studies of the role of miR34A in cellular senescence have shown that miR34A may be activated independently of p53 to inhibit MYC in an alternative pathway that involves the ETS transcription factor ELK1 [17] (Figure 5). This is in support of the current observations, which show that patients with “double-hit” DLBCL have a particularly poor outcome.

Treatment of tumors with loss of p53 is challenging since conventional chemotherapy targets both proliferating normal cells and cancer cells. One promising new treatment strategy is cyclotherapy, which allows targeting p53 deficient cancer cells while shielding normal cells with intact p53 from cytotoxicity [51–53]. Alternative treatment options include miR34 mimics, which retain apoptotic pathways in p53 deficient cancer cells. It has been demonstrated that the restoration of miR34A expression can inhibit the growth of *TP53*-mutant gastric carcinoma cells [54], and that miR34A mimics inhibit the growth of DLBCL and pulmonary carcinoma in *in vivo* mouse models [38, 55]. Interestingly, a recent study showed that miR34A may activate p21 downstream of p53 by downregulation of HDAC1 [56]. However, an important limitation to *in vivo* pharmacological targeting of miRs is systemic delivery, although novel advances such as lipophilic nanoparticles are promising. [57]. An additional concern is that the function of individual miRs may be dependent on the particular cellular context. Anti-miR34s have been shown to improve myocardial function [58], thus overexpression may lead to unforeseen side effects. However, for miR34A these obstacles may be overcome: Studies of many different cancer cell types including the present study suggest that miR34s for the larger part are downregulated by promoter hypermethylation, and we show that miR34A-5p can be upregulated by a demethylating agent in DLBCL cells with a methylated *MIR34A* promoter in cells with and without *TP53* mutations.

Thus we believe, we have identified a novel rare, aggressive “double-hit” DLBCL that may be targeted by demethylating therapy prior to or in combination with conventional immunochemotherapy.

## MATERIALS AND METHODS

### Specimens

Pre-treatment DLBCL biopsies were obtained from 150 patients, and reactive lymph nodes from 6 individuals with inflammatory disease and CD19+ peripheral blood B-lymphocytes (PBL-B), and CD3+T-cells from 6 healthy donors were used as controls. All patients were treated with anthracycline containing regimens (CHOP or CHOP-like). Sixty-two of the patients also received Rituximab (R). The clinical data were obtained from the patient files and from the Danish lymphoma registry, LYFO. Approval of this study was obtained from the ethical committee.

### *In vitro* activation of normal B-cells

PBL-B purified from healthy donors by RoboSep human CD19 positive selection kit (Stemcell) were plated 5 x 10^5^ cells/ml and cultured in 20% HIFCS RPMI1640 with 100 units/ml penicillin, 100μg/ml streptomycin, 50 ug/ml lipopolysaccarides from Escherichia coli O55:B5 (LPS E.coli, Sigma Aldrich), and 0,6 ng/mL IL-1α (Genescript) to stimulate IgM and IgG3. The activated B cells were harvested on day 4.

### Cell lines and treatment

DLBCL cell lines: DB1, Toledo, Farage, DOHH2, HT, and U2932 were cultured as above. HT and U2932 cells were seeded 2x10^5^ cells/ml 24 hrs prior to treatment with 0.1μM and 0.5 μM of the DNA methyltransfrease inhibitor 5-Aza-2-deoxycytidine (5-Aza-CdR;Sigma-Aldrich). The medium was changed and the drug removed after 24 hrs treatment, and cells were collected 1 day and 5 days later. Untreated cells and cells treated with cytosine arabinoside (AraC; Sigma-Aldrich) 20nM or 100nM were grown and harvested under similar conditions as control.

### MIR34A and MIR34B/C chromatin immunoprecipitation (ChIP)

PBL-B were crosslinked, nuclear extracts sonicated, and mixed with 10 μg of antibodies H3 (Abcam ab791), H3K27me3 (Millipore 17-622) and 4 μg of antibody H3K4me3 (Active Motif 39159). Parallel preimmune control precipitation was performed by normal IgG (Cell Signaling 2729). DNA precipitated in the ChIP experiments was amplified by RT-qPCR at the promotors, transcription start sites (TSS) and genomic regions for *MIR34A* and *MIR34B/*C using the primers in Supplemental Table 1 a/b.

### Detection of TP53 mutations

The coding sequences and splice sites of exons 5-9 of the *TP53* gene were scanned for mutations by PCR and denaturing gradient gel electrophoresis (DGGE) as desribed [59]. All mutations were confirmed in a second round of PCR from the original sample.

### Promoter hypermethylation of MIR34A and MIR34B/C

One μg of each DNA sample was bisulfite converted using the EZ DNA Methylation kit (Zymo Research). The methylation status of the *MIR34A* and the *MIR34B/C* promoters was examined using methylation-specific melting curve analysis (MS-MCA) [60]. The DNA sequences analyzed for promoter hypermethylation locate to the promoter CpG islands at the TSSs as identified by others [33, 61]. For both segments, amplification was carried out using the LightCycler 480 instrument (Roche diagnostics) as described [62]. For primers see Supplemental Table 1c. The melting peaks were calculated using the LightCycler 480 Software Release 1.5.0SP3. *SssI* treated DNA (Millipore) was bisulfite converted and used as positive control. Bisulfite converted DNA from PBL-B was used as normal control.

### Bisulfite sequencing

To analyze the methylation status of individual *MIR34A* alleles PCR products were cloned into the pCR2.1 vector using the TOPO-TA cloning kit (Invitrogen). Colonies were screened for the respective inserts, amplified using TempliPhi (Amersham Biosciences) and 10 individual colonies were sequenced. For primers see Supplemental Table 1d.

### MiR34A, miR34B, and miR34C expression

Fifty ng of RNA was reverse transcribed (RT) using miRCURY LNA™ Universal RT (Exiqon). RT-qPCR for miR34A-5p, miR34A-3p, miR34B-5p, miR34B-3p, miR34C-5p and miR34C-3p was performed using miRCURY LNA™ Universal RT primer sets (nomenclature according to miRbase18), and U44 RNA was used for normalization (prod. No. 204486, 204318, 204005, 204424, 204373, 204407, 203902, respectively). The RT-qPCRs were performed on the LightCycler 480 instrument (Roche diagnostics) using the conditions recommended by Exiqon. Absolute quantification was performed using a miR-34A-5p mimic in final amounts of 1x10^8^ copies to 10 copies per RT reaction.

### Statistics

Differences in clinical characteristics of patients with or without methylation of *MIR34A, MIR34B/C* and mutation of *TP53* were assessed using the Pearson chi-square or Fisher's exact tests. Overall survival was estimated using the Kaplan-Meier method and log-rank test. For assessment of independent predictors of survival a multivariate Cox regression hazard model with backward stepwise (likelihood ratio) entry was applied. Effects not meeting a p-value < 0.05 were removed from the model. Statistical analyses were performed in SPSS 19.0 for Windows (SPSS Inc.) and SAS version 9.20 (Cary, NC). Any differences were considered to be statistically significant when the p-value was <0.05.

## SUPPLEMENTARY FIGURES AND TABLES


