# Supplementary material for: Diffuse large B-cell lymphoma with combined TP53 mutation and MIR34A methylation: Another “double hit” lymphoma with very poor outcome?
Source: Oncotarget. 2014 Mar 31;5(7):1912–25. doi: 10.18632/oncotarget.1877 (PMC4039115; doi:10.18632/oncotarget.1877)
Supplement: Supplementary file 1 [file oncotarget-05-1912-s001.pdf]

Supplemental Information

Analysis of the role of *MDM2*-SNP309 and *MIR34A* sequence variants in de novo DLBCL

In previously untreated CLL it has been shown that cases that carry the MDM2-SNP309 GG alleles have downregulation of miR34a, indicating that the GG-genotype could phenocopy p53 loss.[20] Therefore we analysed the in MDM2 SNP309 in 120 of the 150 cases. In addition, it was previously shown that MIR34a carry polymorphisms *(www.genome.ucsc.edu)*, so we decided to analyse the cases? for polymorphisms in these two genes in 120 of the 150 DLBCL cases.

METHODS

***MDM2* SNP 309**

SNP309 genotyping was done in 120 of the cases using fluorescent Taqman probes to discriminate between the common SNP309 genotype (T/T) and the variant alleles (T/G and G/G). Briefly, sense primer (MDM2-F, 5´-GGAGTTCAGGGTAAAGGTCACG-3´), antisense primer (MDM2 AS (5´-GCGCAGCGTTCACACTAGTG-3´), and the polymorphism-specific hydrolysis probes MDM2-T-FAM (5´-CCGCT**T** CGGCGC G-3´) and MDM2-G-VIC (5´-CCGCT**G** CGGCGC G-3´, were used. For PCR, UniversalPCRMaster mix, AmpErase UNG (Applied Biosystems, Foster City, CA), primers, and 10 ng of DNA were mixed. Conditions for PCR were as follows: 95°C (10 min) and 40 cycles of (95°C (15 sec), 60°C (60 sec)). Amplification was performed using LightCycler 480 and analyzed using endpoint genotyping in the LightCycler 480 software package.
in RPMI 1640 medium with Glutamax-1 plus 10% FBS. 100 units/ml penicillin and 100µg/ml streptomycin was added to the cultures.

Detection of *MIR34A* sequence variants.

The MIR34A coding sequence was amplified using the primers [40CG-clamp]-5'-GCAGCACTTCTAGGGCAGTATACTTG-3' and 5'-GGACCGGCCAGCTGTGAG-3', and run at 55°C in a gradient gel containing 10-70% denaturant. Abnormal DGGE bands were excised from the gels, the DNA eluted in water, reamplified, and automatically sequenced (Eurofins, MWG, Germany).

RESULTS

The GG variant of MDM2 SNP309 in DLBCL

Since studies in CLL suggest that the *MDM2* SNP309 GG genotype associate with low miR34a expression and poor prognosis, we investigated the role of this SNP in 120 primary DLBCLs. Eleven of 120 DLBCLs (9%) carried the *MDM2* SNP309 GG-genotype, one GG case also carried a *TP53* mutation (p=0.69), and another GG case carried concomitant methylation of *MIR34A* and *MIR34B/C* (p=0.18), indicating that *TP53* mutation and *MIR34A/B/C* methylation occur independent of the MDM2 SNP309 GG-genotype. No differences in survival were observed for cases with the *MDM2* SNP309 GG-genotype compared to GT/TT genotypes (p=0.88). Two cases with combined GG-genotype and *MIR34A/B/C* methylation or *TP53* mutation did not have a shortened survival compared to "single-hit" cases.

MIR34A sequence variation in DLBCL.

No sequence variants were identified in the *MIR34A* sequence, although 2 SNPs have previously been reported in the region.

**Table S1a: Primers for ChIP RT-qPCR *MIR34A***			
Location on Chr 1	Forward primer sequence (5’→3’)	Reverse primer sequence (5’→3’)	Product length (bp)
Promotor 9.242.434-9.242.211	GACGTGATTCGGATCGCGC	CAGGCAAGCCCAGGCAGAG	223
Downstream promoter 9.242.143-9.241.947	GTCCTGCAGCCAAGCTCCG	GAGGCTACACAATTGAACAGGGT	197
MIR34A 9.211.836-9.211.727	GGACCGGCCAGCTGTGAG	GCAGCACTTCTAGGGCAGTATACTTG	101

**Table S1b: Primers for ChIP qRT-PCR *MIR34B/C***			
Location on Chr 11	Forward primer sequence (5’→3’)	Reverse primer sequence (5’→3’)	Product length (bp)
Promotor 111,383,203-111,383,341	ACAGTCACTCGGCCGCTCAG	CTCGCTTTTCCTTTCCCCTTC	139
MIR34B 111,383,682-111,383,736	GTGCTCGGTTTGTAGGCAG	CGGTGATGCTGTGCCTTGTT	94
MIR34C 111,384,164-111,384,240	TGAGACTGCAATTTTTTCTATGAGT	CATTGATGATGCACAGGCAG	139

**Table S1c: Primers for MS-MCA**			
	Forward primer sequence (5’→3’)	Reverse primer sequence (5’→3’)	Product length (bp)
MIR34A	TTTTTTTTTAGGTGGAGGAGATGT	CCAAACAAACCCAAACAAAAC	155
MIR34B/C	TTGTTATTAAAATAAGGTATAGTATT A	CGCTTCTCAAACATCTTCTCT	99

For *MIR34A* the amplified region includes 14 CpG sites located at the initiation site and overlapping the p53 binding motif. For *MIR34B/C* the amplified region includes 10 CpG sites of the 5’CpG island located in the common *MIR34B/C* promoter.

**Table S1d: Primers for bisulfite sequencing**			
	Forward primer sequence (5’→3’)	Reverse primer sequence (5’→3’)	Product length (bp)
MIR34A	AGGGGATGAGGATTAGGATTT	CCAAACAAACCCAAACAAAAC	347

**Table S1e: Primers for *MIR34A* mutation analysis by DGGE**			
	Forward primer sequence (5’→3’)	Reverse primer sequence (5’→3’)	Product length (bp)
MIR34A	[40CG-clamp]-GCAGCACTTCTAGGGCAGTATACTTG	GGACCGGCCAGCTGTGAG	141

Supplemental Table 2

Number	SNP309	*RB4A*	*RB4B* /C	*TP53* mutation
1	T/G	METH	METH	c.559 +1G>A, splice defect
2	T/T	METH	METH	GGC>AGC; G244S
3	T/G	METH	METH	c.610_612 ddGAG
4	T/G	METH	METH	TGC>TAC;C141Y
5		METH	Unmeth	CCC>CTC; P151L
6		METH	METH	CCC>CTC; P151L
7	T/T	METH	METH	CGA>TGA;R196stop
8	T/T	METH	METH	CCC>CAC;P151H
9	T/T	METH	METH	CAG>TAG; Q192stop
10	G/G	METH	METH	
11	T/T	METH	METH	
12	T/G	METH	METH	
13	T/T	METH	METH	
14	T/T	METH	METH	
15	T/G	METH	METH	
16		METH	METH	
17		METH	METH	
18		METH	METH	
19		METH	METH	
20	T/G	METH	METH	
21	T/G	METH	METH	
22	T/T	METH	METH	
23		METH	METH	
24	T/T	METH	METH	
25	T/G	METH	METH	
26	T/T	METH	METH	
27	T/G	METH	METH	CGA>CGG; R213R SNP
28	T/T	METH	METH	
29	T/G	METH	METH	
30	T/T	METH	METH	
31	T/G	METH	METH	
32	T/T	METH	METH	
33	T/G	METH	METH	
34	T/G	METH	METH	
35	T/T	METH	.	
36	T/G	METH	METH	
37	T/T	METH	Unmeth	
38	T/T	METH	METH	
39	T/G	METH	METH	
40	T/T	METH	METH	
41	T/T	METH	METH	
42	T/T	METH	METH	

43	T/T	Unmeth	Unmeth	AAG>CAG; K164E
44	T/G	Unmeth	METH	c521-539 del18bp fs and intron 6+31G>A
45		Unmeth	METH	TAC>TGC; Y234C
46		Unmeth	METH	CAC>CAA; H167Q
47	G/G	Unmeth	METH	CGG>TGG; R248W
48	T/T	Unmeth	METH	CGC>CAC; R175H
49	T/T	Unmeth	METH	CGC>CAC; R175H
50	T/T	Unmeth	METH	GAC>AAC; D259N
51	T/T	Unmeth	METH	CGG>TGG; R273W and AGT>AAT; S215N
52	T/G	Unmeth	METH	c.869+31A>G
53	T/G	Unmeth	METH	CTT>CCT; L194P
54	T/T	Unmeth	METH	CGG>CAG; R248Q
55	T/T	Unmeth	Unmeth	CAT>CGT; H193R
56	T/G	Unmeth	Unmeth	TGC>TAC; C176Y
57	G/G	Unmeth	METH	CGC>CAC; R175H
58	T/T	Unmeth	METH	CGT>TGT; R273C
59	T/T	Unmeth	Unmeth	
60	T/T	Unmeth	METH	
61	T/T	Unmeth	METH	
62	T/T	Unmeth	Unmeth	
63	G/G	Unmeth	METH	
64	T/G	Unmeth	METH	
65	T/G	Unmeth	METH	
66	T/T	Unmeth	METH	
67	T/T	Unmeth	METH	CGA>CGG; R213R SNP
68	T/G	Unmeth	METH	
69	T/T	Unmeth	Unmeth	CGA>CGG; R213R SNP
70	G/G	Unmeth	METH	
71	T/T	Unmeth	Unmeth	
72	T/G	Unmeth	METH	
73	T/T	Unmeth	METH	
74		Unmeth	METH	
75		Unmeth	METH	
76		Unmeth	METH	
77		Unmeth	METH	
78		Unmeth	METH	
79		Unmeth	METH	
80		Unmeth	Unmeth	
81		Unmeth		
82		Unmeth	METH	
83		Unmeth	METH	
84		Unmeth	METH	
85		Unmeth	METH	
86		Unmeth	Unmeth	

87		Unmeth	METH	
88		Unmeth	METH	
89		Unmeth	METH	
90		Unmeth	mangle	
91		Unmeth	Unmeth	
92		Unmeth	Unmeth	
93		Unmeth	METH	
94		Unmeth	METH	
95		Unmeth	METH	
96	T/G	Unmeth	Unmeth	
97	T/T	Unmeth	METH	
98	T/G	Unmeth	METH	
99	G/G	Unmeth	METH	
100	T/T	Unmeth	METH	
101	T/G	Unmeth	Unmeth	
102	T/T	Unmeth	METH	CGA>CGG; R213R SNP
103	T/T	Unmeth	Unmeth	
104	T/G	Unmeth	Unmeth	
105	T/T	Unmeth	METH	
106	T/T	Unmeth	Unmeth	
107	T/G	Unmeth	Unmeth	
108	T/G	Unmeth	METH	
109	T/T	Unmeth	METH	
110	T/G	Unmeth	METH	
111	T/T	Unmeth	METH	
112	T/G	Unmeth	METH	
113	T/G	Unmeth	METH	c.993+12T>C polymorphism
114	T/T	Unmeth	Unmeth	CGG213 polymorphism
115	T/T	Unmeth	METH	
116	T/T	Unmeth	Unmeth	
117	T/T	Unmeth	Unmeth	
118	T/T	Unmeth	METH	
119	T/G	Unmeth	METH	
120	T/G	Unmeth	METH	
121	T/T	Unmeth	METH	
122	T/G	Unmeth	METH	
123	G/G	Unmeth	Unmeth	
124	T/G	Unmeth	METH	
125	T/G	Unmeth	METH	
126	G/G	Unmeth	METH	
127	T/T	Unmeth	Unmeth	
128	T/G	Unmeth	METH	
129	T/T	Unmeth	Unmeth	
130	T/T	Unmeth	METH	

131	T/T	Unmeth	METH
132	T/G	Unmeth	Unmeth
133	T/T	Unmeth	METH
134	T/G	Unmeth	METH
135	T/T	Unmeth	METH
136	G/G	Unmeth	Unmeth
137	T/G	Unmeth	Unmeth
138	T/T	Unmeth	METH
139	T/T	Unmeth	METH
140	T/T	Unmeth	Unmeth
141	T/T	Unmeth	METH
142	T/G	Unmeth	METH
143	T/T	Unmeth	METH
144	G/G	Unmeth	METH
145	T/G	Unmeth	Unmeth
146	G/G	Unmeth	Unmeth
147	T/T	Unmeth	METH
148	T/T	Unmeth	METH
149	T/G	Unmeth	METH
150	T/T	Unmeth	Unmeth

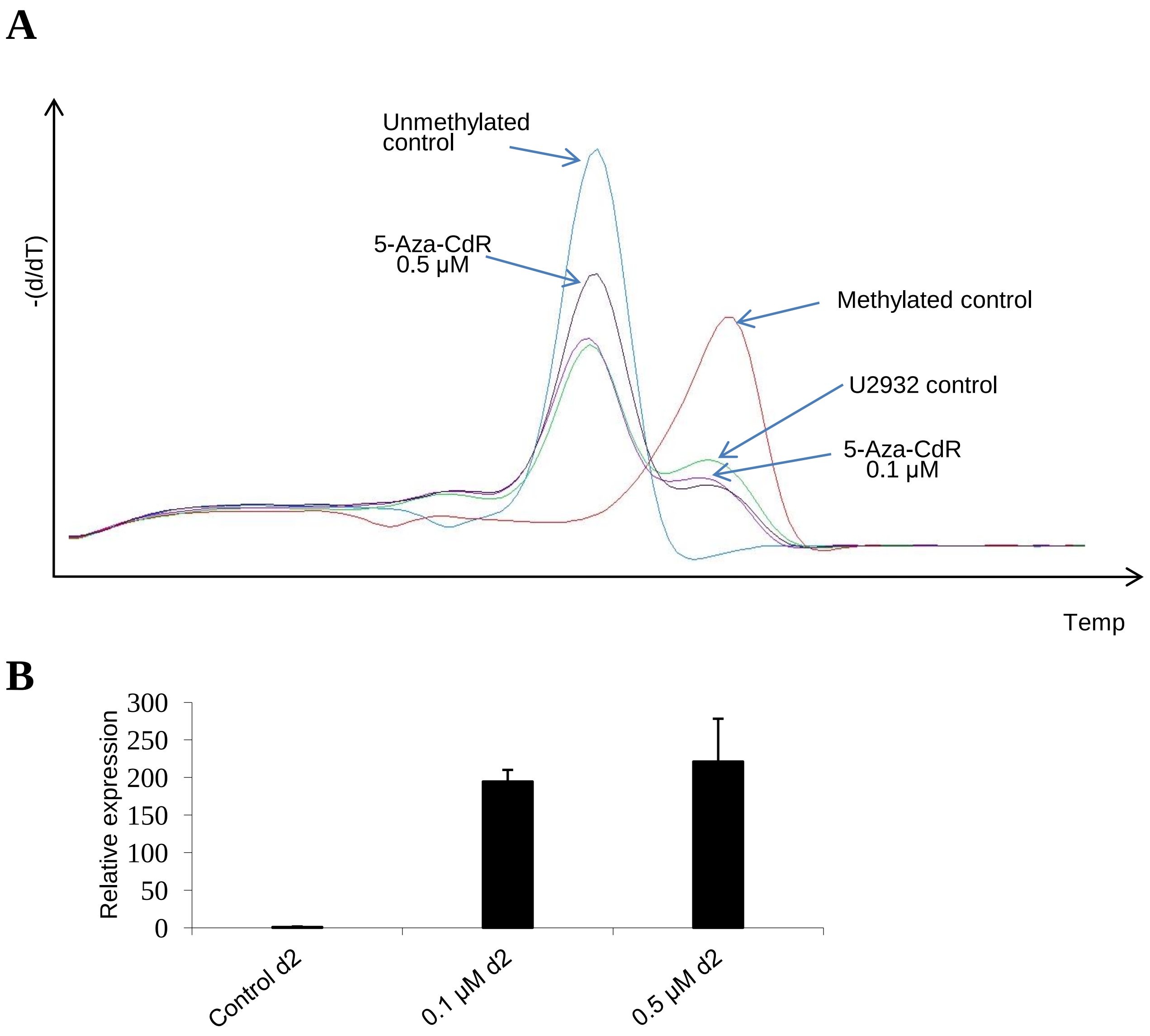

Supplemental Figure 1. The expression of miR34a in the *MIR34A* methylated and *TP53* mutated cell line, U2932 is regulated by promotor methylation. U2932 was treated with 5-aza-2’deoxycytidine 0.1 and 0.5 µM resulting in (A) decrease in the methylated peak and an increase in the unmethylated peak observed by Ms-MCA and (B) induction of the expression of miR-34a relative to the untreated control.

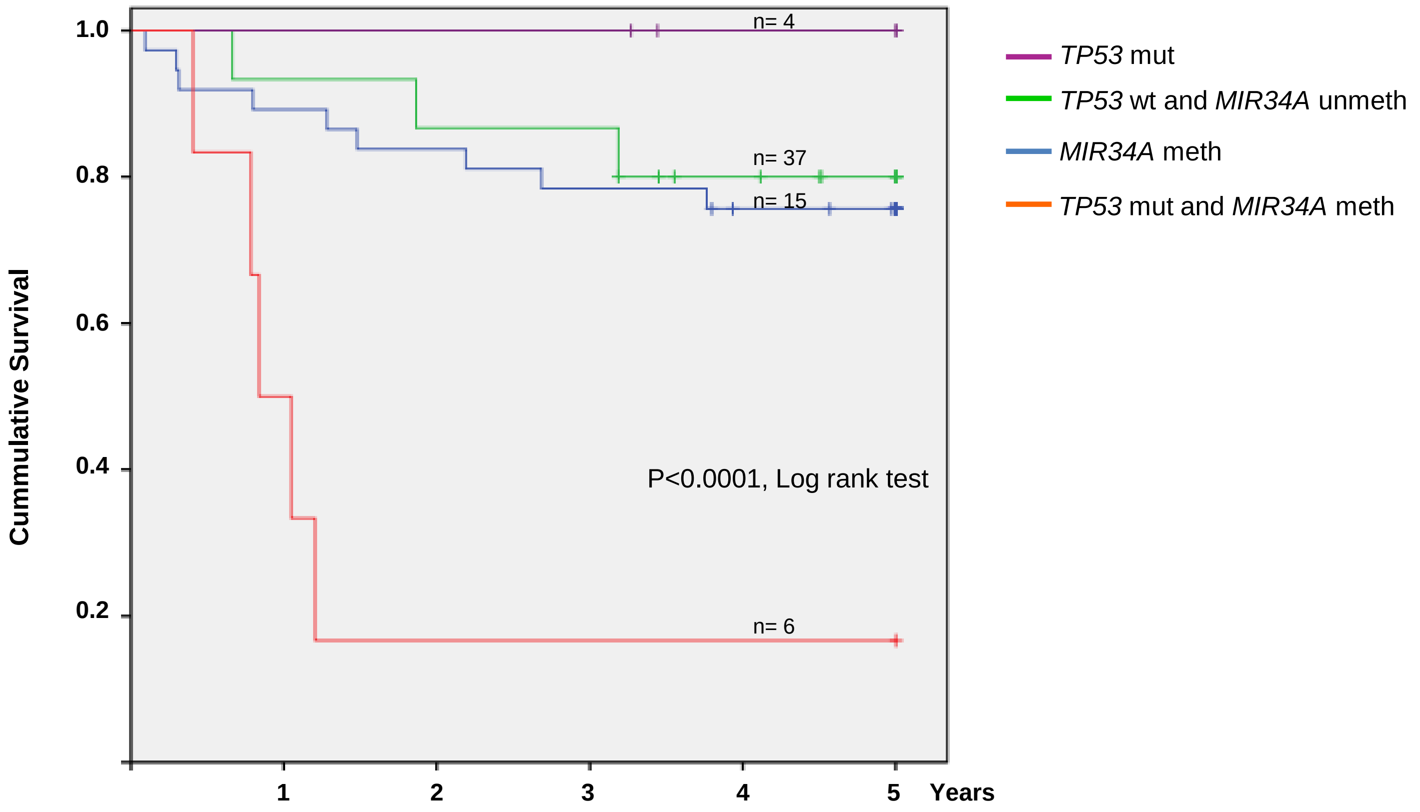

Supplemental Figure 2. 5 year overall survival for the patients treated with Rituximab (n=62) stratified according to *TP53*wt and unmethylated *MIR34A* unmethylation, *TP53* mutation alone, *MIR34A* methylation alone, and *TP53* mutation and *MIR34A* methylation status. The group with concomitant *MIR34A* methylation and *TP53* mutation (n=6) shows significantly poor overall survival (*P*<0.0001), and none of the patients with *TP53* mutation alone (n= 4) died in the follow-up time more than 3 years.

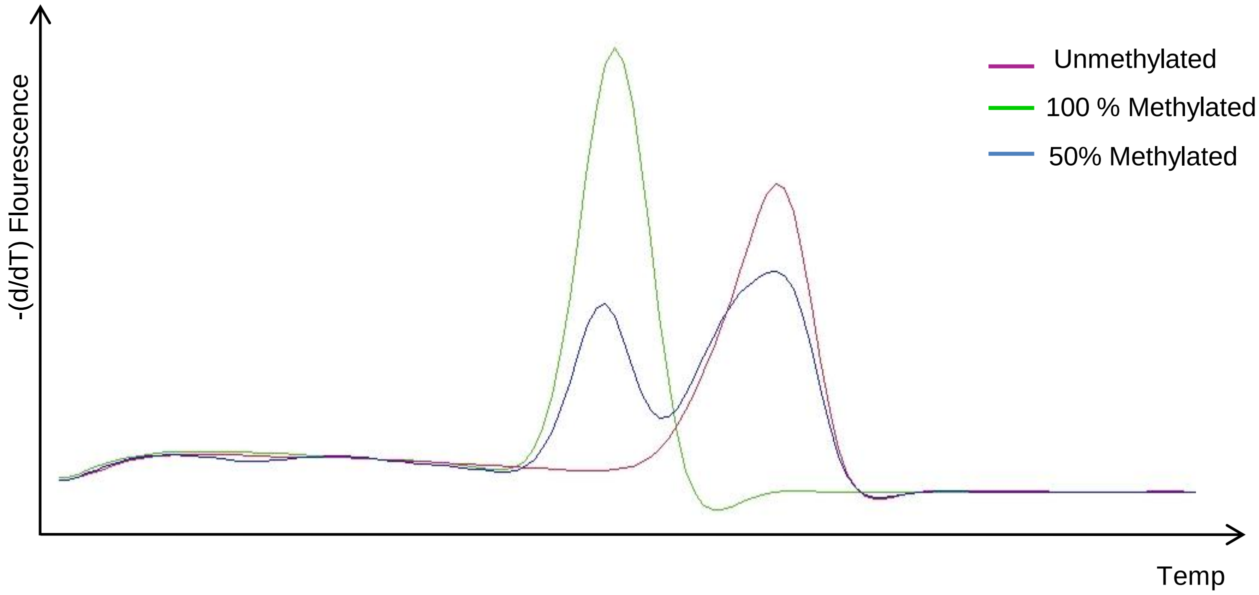

Supplemental Figure 3. MsMCA of *MIR34A* promotor. showing 100%, 50% methylation and no methylation.
